# The Medical Aspects of Religious Revivals

**Published:** 1905-02-11

**Authors:** 


### The Hospital.

A Journal of

The Medical Sciences and Hospital Administration.

Vol. XXXVII.—No. 959. FEBRUARY 11, 1905.

### The Medical Aspects of Religious Revivals.

The so-called "revival" of religion, which is now taking place in Wales and also in connection with the American missioners in London is a matter upon the theological merits or demerits of which we have no desire to pronounce, but which is practically certain to be attended by phenomena specially interesting to the medical profession. These phenomena, in slightly varying forms, have repeated themselves in many countries and on many different occasions. During the 18th and the earlier part of the 19th century (without mentioning many examples in more remote times) epidemics of a more or less convulsive character have been brought about by preaching, epidemics of which no precise or strictly medical history has been handed down, but which, as far as can be judged from the scanty records preserved of them, appear to have been much alike in all their essential features. A very remarkable "revival" was that which occurred in Belfast in the beginning of 1859, and which was made the subject of a highly interesting and instructive pamphlet by the Rev. Edward Stopford, Archdeacon of Meath, who endeavoured to explain the possibility of establishing a dividing line between the beneficial effects of awakened religious feeling, and the rhapsodies of hysterical or cataleptic ecstasy. According to the archdeacon's description, catalepsy, and cataleptic ecstasy were matters of frequent occurrence at certain places of worship, and, when he first wrote, were daily on the increase. Mill girls were praying to be "struck." The best "cases" were obtaining a comfortable maintenance by relating their "visions" to daily levées of admiring auditors. The clergy, of all denominations, were wholly unprepared for the emergency, were ignorant, speaking generally, of what it was they witnessed, and do not appear to have imagined that medical men could have any special knowledge of the causes in operation, or any special duties with regard to the effects produced. Indeed, in words which the archdeacon quoted from a correspondent, all the pious ladies of Belfast would have combined to destroy the practice of a doctor who had expressed any doubt of the divine origin of the prevailing manifestations.

Archdeacon Stopford had the discernment to perceive the nature of the physical effects produced by the preaching, and the courage to call these effects by their right names. He traced some of the attacks of hysteria to the abuse of certain pulpit arts, to the reiteration of hell! hell! hell! and to the consequent accumulation among the congregations of depressing emotions for which no adequate outlet was provided in active thought or in practical duty. He traced others to the kindred but more gradual operation of the highly charged mental atmosphere of the locality, and he described the nature and tendencies of the induced disease with sufficient clearness to disabuse all candid minds of the supposition that it could ever, in any circumstances, be rendered an instrument of good. Physiologists, and physicians as physiologists, are bound to recognise that emotion is a force seeking outlet in action, capable of being guided by those who have been trained to bring it into subjection, but certain, when suffered to accumulate, to overpower persons of feeble will, and to compel them into courses which sound judgment would often be unable to approve. Abandonment to religious feeling, like abandonment to any other feeling, is merely a surrender of the will to the emotions, and its principal effect is to render emotion, and not will, the predominant factor in the organism.

We may, if we please, call the religious emotion a "good" one, and we may perhaps recognise its feeblest form in the case of those ladies who say that they always feel "good" in a cathedral. Subdued light, and magnificent architecture, and devotional music, appeal to their feelings in some transient fashion; but they return to ordinary worldliness as they step out into the air. The "cases" of the revivalist differ from these in the greater force of the original emotion, and in the extent to which it is maintained by surrounding circumstances and restrained from immediate escape in action. The subjects of it do not step out into the air; and the force which has been excited obtains no immediate channel of operation, but is restrained until it produces an explosion. The immediate effects of the explosion may be various, the ultimate effect is always the same. The subject has been taught to brood over mere feeling until it masters the will, and thus at first weakens, and ultimately annihilates, the power of self control. The religious emotion may appear to some people to be good and laudable, but it is in its nature transient, and the supremacy which it has obtained only paves the way for the supremacy of any other emotion by which it may be succeeded. This, in many instances, and especially in congregations of young people brought together to be excited by preaching, is very frequently an emotion of sex; and the records of many past revivals are deeply stained by the profligacy which has been the immediate consequence of the hysteria.

We do not doubt that the revivalist meetings will be crowded. Any *nouveau frisson*, to use the slang of the day, is certain to appeal to idle and brainless people of fashion, and any excitement will be attractive to Lisa of Lambeth. It may none the less be the duty of many medical practitioners to speak a word of warning in due season, and to point out the evil tendencies of emotional excitement, to whatever causes it may be attributable, or by whatever names it may be called. The "religion" described by the apostle, "to visit the fatherless and widows in their affliction, and to keep himself unspotted from the world," is of a type distinctly superior to that which is said to be "got" at camp meetings and revivals, however well intentioned may be their leaders and promoters.

